# Fetal Exposure to Maternal Smoking and Neonatal Metabolite Profiles

**DOI:** 10.3390/metabo12111101

**Published:** 2022-11-11

**Authors:** Kim N. Cajachagua-Torres, Sophia M. Blaauwendraad, Hanan El Marroun, Hans Demmelmair, Berthold Koletzko, Romy Gaillard, Vincent W. V. Jaddoe

**Affiliations:** 1 The Generation R Study Group, Erasmus MC, University Medical Center Rotterdam, 3000 CA Rotterdam, The Netherlands; 2 The Department of Pediatrics, Erasmus MC, University Medical Center Rotterdam, 3000 CA Rotterdam, The Netherlands; 3 The Department of Child and Adolescent Psychiatry, Erasmus MC, Sophia Children’s Hospital, 3000 CB Rotterdam, The Netherlands; 4 The Department of Psychology, Education and Child Studies, Erasmus School of Social and Behavioural Sciences, 3062 PA Rotterdam, The Netherlands; 5 Department of Pediatrics, Dr. von Huaner Children’s Hospital, LMU University Hospitals, LMU—Ludwig Maximilians Universität Munich, 80539 Munich, Germany

**Keywords:** smoking, pregnancy, neonatal, metabolite profile, metabolism

## Abstract

Fetal tobacco exposure has persistent effects on growth and metabolism. The underlying mechanisms of these relationships are yet unknown. We investigated the associations of fetal exposure to maternal smoking with neonatal metabolite profiles. In a population-based cohort study among 828 mother-infant pairs, we assessed maternal tobacco use by questionnaire. Metabolite concentrations of amino acids, non-esterified fatty acids, phospholipids and carnitines were determined by using LC-MS/MS in cord blood samples. Metabolite ratios reflecting metabolic pathways were computed. Compared to non-exposed neonates, those exposed to first trimester only tobacco smoking had lower neonatal mono-unsaturated acyl-alkyl-phosphatidylcholines (PC.ae) and alkyl-lysophosphatidylcholines (Lyso.PC.e) 18:0 concentrations. Neonates exposed to continued tobacco smoking during pregnancy had lower neonatal mono-unsaturated acyl-lysophosphatidylcholines (Lyso.PC.a), Lyso.PC.e.16:0 and Lyso.PC.e.18:1 concentration (False discovery rate (FDR) *p*-values < 0.05). Dose-response associations showed the strongest effect estimates in neonates whose mothers continued smoking ≥5 cigarettes per day (FDR *p*-values < 0.05). Furthermore, smoking during the first trimester only was associated with altered neonatal metabolite ratios involved in the Krebs cycle and oxidative stress, whereas continued smoking during pregnancy was associated with inflammatory, transsulfuration, and insulin resistance markers (*p*-value < 0.05). Thus, fetal tobacco exposure seems associated with neonatal metabolite profile adaptations. Whether these changes relate to later life metabolic health should be studied further.

## 1. Introduction

Tobacco smoking during pregnancy is a major clinical and public health problem worldwide [1]. Smoking during pregnancy is associated with various pregnancy complications such as preterm birth, low birth weight and small size for gestational age (SGA) [2,3,4,5]. Also, previous studies have suggested that fetal exposure to tobacco smoke is associated with obesity, hypertension and impaired glucose tolerance in children and adults [4,6,7,8,9,10]. The developmental programming mechanisms by which fetal tobacco exposure leads to impaired cardio-metabolic health might include the disruption of fetal metabolic pathways. Metabolomics has become a useful tool to identify yet unknown molecular pathways. Studies in adults showed that smoking is associated with reduced acyl-alkyl-phosphatidylcholines (PC.ae) and increased diacyl-phosphatidylcholines (PC.aa) concentrations in blood serums reflecting altered lipid metabolism [11,12]. These studies showed sex-specific effects of smoking on the adult metabolome profile [11,12]. A small study among 40 full-term newborns showed that maternal smoking in pregnancy may lead to lower cord blood acyl-carnitine (Carn.a) concentrations and higher lipids concentrations, such as phosphatidylcholine (PC) and sphingomyelin (SM) [13]. In addition to this, a growing body of literature suggests sex-specific differences in fetal programming in response to environmental stress, which may result in sex-specific risk for later disease [5,6,7,8,9,10,14,15,16,17,18]. We hypothesized that exposure to tobacco smoke during pregnancy, both in the first trimester only and throughout the pregnancy, leads to metabolic adaptations in the unborn child, which might predispose individuals to cardio-metabolic disease in later life. Insight into the metabolic developmental adaptations in response to fetal tobacco exposure may contribute to an understanding of pathways in early life that predispose to cardiovascular and metabolic disease in later life.

We examined the associations of fetal tobacco exposure with neonatal metabolite profiles from umbilical cord blood in a population-based cohort among 828 children. We also explored whether any sex specific associations exist.

## 2. Material and Methods

### 2.1. Study Design

This study was embedded in the Generation R Study, a population-based on a prospective cohort study from fetal life until adulthood in Rotterdam, the Netherlands [19]. The study was approved by the Medical Ethics Committee of the Erasmus Medical Center in Rotterdam, the Netherlands (MEC 198.782/200/31, 17 December 2001). Written informed consent was obtained from all participants. Pregnant women living in the study area with an expected date of delivery between April 2002 and January 2006 were recruited. In total, 8879 mothers (response rate of 61%) were enrolled during pregnancy. The current study was performed in a random group of 1232 mothers and their children of Dutch ethnicity, which was defined at the start the cohort study [20]. Dutch ethnicity was defined as having both parents born in the Netherlands based on the classification of Statistics Netherlands [21]. Of these, 1103 mothers had information on tobacco use during pregnancy and had singleton live births. Cord blood samples for metabolomics analyses were obtained at birth among 828 live-born children (Figure S1).

### 2.2. Fetal Tobacco Exposure

As previously described, information on maternal smoking was collected by postal questionnaires in early pregnancy (median 12.9 weeks of gestation, 25–75th percentiles 12.1–14.5), mid-pregnancy (median 20.4 weeks of gestation, 25–75th percentiles 20.4–20.9), and late pregnancy (median 30.2 weeks of gestation, 25–75th percentiles 29.9–30.8) [22]. In early pregnancy, mothers were asked whether they smoked during pregnancy. Then, in mid- and late pregnancy, mothers were asked whether they smoked in the last 2 months. We grouped mothers into three categories: never smoked, smoked until pregnancy was known (first trimester only), and continued smoking during pregnancy. Mothers who reported in the first questionnaire that they smoked during the first trimester only, but still reported smoking in the second and third questionnaire were reclassified into the ‘continued smoking’ category. The same strategy was used for mothers who reported no smoking in the first questionnaire, but reported smoking in the second or third questionnaire. The information on maternal frequency of cigarettes smoked daily was collected in the following six categories (<1 per day, 1–2 per day, 3–4 per day, 5–9 per day, 10–19 per day, and ≥20 per day). These original categories were combined and reclassified into the following categories: no, <5 per day, and ≥5 per day. These categories were created to have enough numbers per category.

Paternal information on tobacco use was assessed by maternal report and paternal self-report during the first trimester of pregnancy. We used maternal reports of paternal tobacco use, given that less fathers (86.4%) completed questionnaires. The inter-rater reliability between maternal and paternal self-reporting was high (Cohen’s kappa = 0.88, *p* < 0.001). Exposure to environmental smoking was assessed in each questionnaire as the number of hours per day that mothers were exposed to environmental smoke at home and at work. For environmental smoking, the following categories were used: no, occasionally, and daily.

### 2.3. Metabolite Measurements

Umbilical venous cord blood samples at birth (median gestational age at birth, 40.4 weeks; 95% range, 37.3–42.3) were collected by midwife and obstetrician, as described previously [23]. The liquid chromatography tandem mass spectrometry (LC-MS/MS) metabolite profiling performed at the Department of Pediatrics, LMU Munich, Germany is described in detail elsewhere [24,25,26]. A targeted metabolomics approach was performed at LMU Munich to determine the serum concentrations (µmol/L) of amino acids, non-esterified fatty acids (NEFA), phospholipids (including PC.aa, PC.ae, acyl-lysophosphatidylcholines (Lyso.PC.a), alkyl-lysophosphatidylcholines (Lyso.PC.e), SM and carnitines (Carn) (including free carnitine (Free Carn) and Carn.a) [20,24,25,27,28]. Detailed information is given in Supplemental Text S1. Briefly, amino acids were analyzed with an 1100 high-performance liquid chromatography (HPLC) system (Agilent, Waldbronn, Germany) coupled to a API2000 tandem mass spectrometer (AB Sciex, Darmsstadt, Germany [25]. IUPAC-IUB nomenclature was used for the notation of amino acids [29]. NEFA, phospholipids and Carn were measured with a 1200 SL HPLC system (Agilent, Waldbronn, Germany) coupled to a 4000QTRAP tandem mass spectrometer from AB Sciex (Darmstadt, Germany) [24,30]. The analytical techniques used are capable of determining the total number of double bonds, but not the position of the double bonds and the distribution of the C-atoms between fatty acid side chains. The following notation was used for NEFA, phospholipids, and Carn.a: ‘X:Y’, where ‘X’ denotes the number of C-atoms of the carbon chains, and ‘Y’ the total number of double bonds. The ‘a’ denotes an acyl chain bound to the backbone via an ester bond (‘acyl-‘), and the ‘e’ represents an ether bond (‘alkyl-’). To assess the precision of the measurement, six quality control (QC) samples per batch were consistently measured between study samples. After exclusion of outliers, the coefficients of variation (CV; standard deviation/mean) for each batch (intra-batch) and for all batches (inter-batch) of the QC samples were calculated for each metabolite. In line with previous studies, for each metabolite we excluded batches with an intra-batch CV higher than 25% [26,31,32,33]. Data on complete metabolites were excluded for metabolites with an inter-batch CV higher than 35%, or if less 50% of the batches passed the QC (i.e., had an intra-batch CV lower than 25%). To correct for batch effects, metabolite concentrations were divided by the ratio of the intra-batch median and the inter-batch median of the QC samples [31]. Metabolites and participants with more than 50% of missing values were excluded [26,31]. Missing metabolite values were imputed using the Random Forest algorithm (R package missForest) [34].

Individual metabolites were clustered in general metabolite groups based on chemical structure (amino acids, NEFA, PC.aa, PC.ae, Lyso.PC.a, Lyso.PC.e, SM, Free Carn, and Carn.a), and in metabolite subgroups based on chemical structure and biological relevance (amino acids: branched-chain amino acids (BCAA), aromatic amino acids (AAA), essential amino acids, non-essential amino acids; NEFA; PC.aa; PC.ae; Lyso.PC.a; Lyso.PC.e; SM: saturated, mono-unsaturated, poly-unsaturated; Carn.a: short-chain, medium-chain, long-chain) [23]. The sum of the individual metabolite concentrations per metabolite group and subgroup were calculated [23]. Since adverse outcomes in offspring due to fetal tobacco exposure may be explained by mitochondrial dysfunction, oxidative stress, immune response, lipid peroxidation, fatty acid-β oxidation and insulin resistance, we computed selected neonatal metabolite ratios reflecting those processes (see Table S1) [35,36,37,38,39,40,41,42].

Individual metabolite concentrations, sums and ratios were square root transformed to normalize metabolite concentrations. To enable comparison of the effect estimates, individual metabolite concentrations and sums and ratios were standardized by calculating standard deviation scores (SDS).

### 2.4. Covariates

Potential covariates were selected based on previous studies and presented as a directed acyclic graphic (Figure S2) [1,10,13,43]. Information on maternal age, education, pre-pregnancy body mass index (BMI), alcohol use and psychopathology score were obtained by self-report questionnaires. Information on education was categorized according to the classification of Netherlands Statistics [44]. Maternal alcohol use was classified according to three categories: never drank in pregnancy; drank in the first trimester only; and continued during pregnancy. Maternal psychopathology was assessed using the Brief Symptom Inventory (BSI) in mid-pregnancy, a validated self-reported measure of 53-items covering a spectrum of psychopathology symptoms [45]. Maternal folate concentrations were measured in blood samples at the first trimester by using an immunoelectrochemoluminiscence assay on the Architect System (Abbott Di-agnostics B.V.). The between-run coefficients of variation were 8.9% at 5.6 nmol/L, 2.5% at 16.6 nmol/L and 1.5% at 33.6 nmol/L, with an analytical range of 1.8–45.3 nmol/L for plasma folate [46]. Information on sex, gestational age at birth, and birth weight were collected from midwives. Birth weight and gestational age were considered as potential mediators. Birth weight was standardized based on gestational age and sex according to European growth charts [47]. SGA was defined as gestational age-adjusted birth weight under the 10th percentile; low birth weight, birth weight less than 2500 g; and preterm was defined as a birth <37 weeks of gestation.

### 2.5. Statistical Analysis

First, we assessed the subject characteristics and performed a non-response analysis by comparing children with and without metabolomics data using a chi-squared test for categorical variables and a Student’s *t*-test or Mann-Whitney U test for continuous variables. Second, we estimated the associations of maternal smoking during pregnancy (categories, dose-response) with neonatal metabolite groups, subgroups and individuals using multiple linear regression models. All models were adjusted for maternal age, education, alcohol use, pre-pregnancy BMI, psychopathology score and fetal sex (confounder model). We examined whether these associations were explained by birth weight or gestational age at birth by additionally adjusting for them, since neonatal metabolic profiles correlate with these birth characteristics (birth outcomes model). To examine potential sex-specific associations, we tested the statistical interaction between maternal smoking and offspring sex, and, if significant interactions were present after taking multiple testing, performed stratified analysis by sex. To account for multiple testing, we applied Benjamin-Hochberg correction using an overall false discovery rate of (FDR) < 5% [48]. Because of the explorative purpose of the analysis of maternal smoking during pregnancy with neonatal metabolite ratios and high correlation between metabolite ratios (Figure S3), nominal *p*-values (*p* < 0.05) were considered in this specific analysis. As sensitivity analyses, first, we used similar analytical approaches to examine the associations of paternal tobacco smoking with neonatal metabolite profiles (excluding mothers who smoked in pregnancy), and the associations of environmental smoking with neonatal metabolite profiles (excluding parents who smoked in pregnancy). Second, we additionally adjusted the associations between fetal tobacco exposure and neonatal metabolite profiles for first trimester blood folate concentrations. The percentage of missingness in the covariates ranged from 0% to 6.5%, with the exception of psychopathology score (12.2%). To account for missing covariates, we used a multiple imputation approach using the multivariate imputation by chained equations (mice) package to estimate missing information of the covariates in 25 datasets and 100 iterations [49]. All statistical analyses were conducted using R statistical software version 3.6.3 (R Foundation for Statistical Computing, Vienna, Austria) [50].

## 3. Results

### 3.1. Characteristics of the Study Population

Of all mothers, 23.8% used tobacco during pregnancy, of whom 9.3% smoked in the first trimester only and 14.5% continued smoking during pregnancy (Table 1). Mothers who continued smoking during pregnancy were younger, less educated, had a higher psychopathology score and lower first trimester blood folate concentrations, and were more often exposed to paternal and environmental smoking compared to mothers who did not smoke. Neonates exposed to continued tobacco smoking during pregnancy had a lower birth weight and a higher risk of SGA as compared to those not exposed. Summed metabolite groups, subgroups and individual concentrations are shown in Table S2. A non-response analysis showed that neonates without information on the neonatal metabolite profile had lower birth weights (Table S3).

### 3.2. Fetal Tobacco Exposure and Neonatal Metabolite Profiles and Ratios

**Figure 1:**
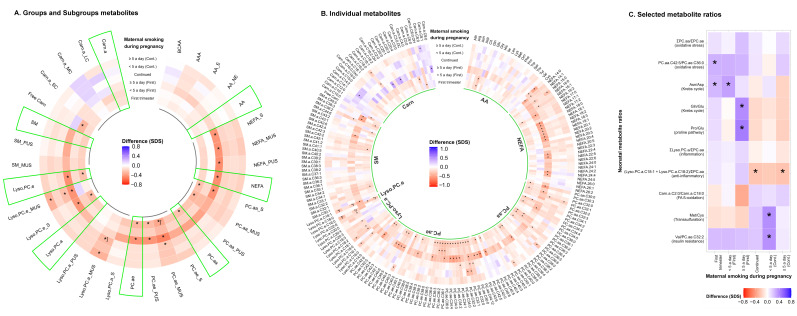
Associations of maternal smoking during pregnancy with cord blood metabolite profiles and ratios. Values represent regression coefficients based on linear models that reflect the positive (blue) and negative (red) difference in neonatal cord blood metabolite concentrations and ratios in standard deviation scores in neonates whose mothers smoked during pregnancy compared to mothers who did not smoke. Models were adjusted for maternal age, educational level, alcohol consumption, pre-pregnancy BMI, psychopathology symptoms, and fetal sex (confounder model). * *p*-value < 0.05. ᶴ Significant associations after FDR corrected *p*-value. The sectors of green color showed group of neonatal metabolite profile, which is preceded by metabolite profile subgroups. Abbreviations: SDS, standard deviation scores; BMI, body mass index; AA, amino acids; BCAA, branched-chain AA; AAA, aromatic AA; AA_E, essential AA; AA_NE, non-essential AA; NEFA, non-esterified fatty acids; NEFA_S, saturated NEFA; NEFA_MUS, mono-unsaturated NEFA; NEFA_PUS, poly-unsaturated NEFA; PC.aa, diacyl-phosphatidylcholines; PC.aa_S, saturated PC.aa; PC.aa_MUS, mono-unsaturated PC.aa; PC.aa_PUS, poly-unsaturated PC.aa; PC.ae, acyl-alkyl-phosphatidylcholines; PC.ae_S, saturated PC.ae; PC.ae_MUS, mono-unsaturated PC.ae; PC.ae_PUS, poly-unsaturated PC.ae; Lyso.PC.a, acyl-lysophosphatidylcholines; Lyso.PC.a_S, saturated Lyso.PC.a; Lyso.PC.a_MUS, mono-unsaturated Lyso.PC.a; Lyso.PC.a_PUS, poly-unsaturated Lyso.PC.a; Lyso.PC.e, alkyl-lysophosphatidylcholines; Lyso.PC.e_S, saturated Lyso.PC.e; Lyso.PC.a_MUS, mono-unsaturated Lyso.PC.e; SM, sphingomyelines; SM_MUS, mono-unsaturated SM; SM_PUS, poly-unsaturated SM; Free Carn, free carnitine; Carn.a, acyl-carnitines; Carn.a _SC, short-chain Carn.a; Carn.a _MC, medium-chain Carn.a; and Carn.a _LC, long-chain Carn.a; Asn/Asp, asparagine/aspartic acid; Gln/Glu, glutamine/glutamic acid; Pro/Glu, proline/glutamic acid; FA β-oxidation, Fatty acid β-oxidation; Met/Cys, methionine/cysteine; and Val, valine.

Maternal smoking during the first trimester only was associated with lower neonatal mono-unsaturated PC.ae and Lyso.PC.e.18:0 concentrations (FDR *p*-values < 0.05) (Figure 1A,B). No dose-response associations were observed between maternal smoking during the first trimester only and any neonatal metabolites (FDR *p*-values < 0.05) (Figure 1A,B). Maternal smoking during the first trimester only was associated with a higher neonatal PC.aa.C42:5/PC.ae.C36:0 and Asn/Asp ratio, but did not survive multiple testing (Figure 1C). Dose-response analyses showed that the strongest association of maternal smoking was <5 cigarettes per day with the Asn/Asp ratio. Also, maternal smoking during the first trimester only ≥5 cigarettes per day was associated with a higher neonatal Gln/Glu and Pro/Glu ratio (Figure 1C). None of the associations were explained by birth outcomes (Figures S4–S6).

Maternal continued smoking during pregnancy was associated with lower neonatal mono-unsaturated Lyso.PC.a, Lyso.PC.e.16:0 and Lyso.PC.e.18:1 concentration (FDR *p*-values < 0.05) (Figure 1A,B). Dose-response associations showed the strongest effect estimates in neonates whose mothers continued smoking ≥5 cigarettes per day (FDR *p*-values < 0.05) (Figure 1B). Maternal continued smoking during pregnancy was associated with a lower Lyso.PC.a.18:1 + Lyso.PC.a.C18:2/∑PC.aa ratio, but did not survive multiple testing (Figure 1C). Maternal continued smoking <5 cigarettes per day was associated with higher neonatal Met/Cys and Val/PC.ae.C32:2 ratios, but did not survive multiple testing. For the Lyso.PC.a.18:1 + Lyso.PC.a.C18:2/∑PC.aa ratio, the strongest effect estimate was observed for maternal continued smoking ≥5 cigarettes per day (Figure 1C). Overall, these associations were not explained by birth outcomes (Figures S4–S6).

### 3.3. Sex-Specific Analyses

**Figure 2:**
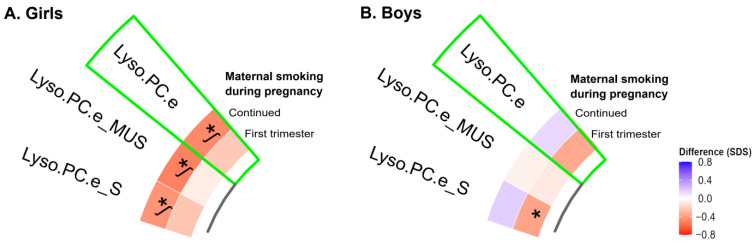
Associations of maternal smoking during pregnancy with cord blood individual metabolite profiles and ratios stratified by sex. Values represent regression coefficients based on linear models that reflect the positive (blue) and negative (red) difference in neonatal cord blood metabolite concentrations in standard deviation scores in neonates whose mothers smoked during pregnancy compared to mothers who did not smoke. Models were adjusted for maternal age, educational level, alcohol consumption, pre-pregnancy BMI, and psychopathology symptoms (confounder model). * *p*-value < 0.05. ᶴ Significant associations after FDR corrected *p*-value. The sectors of green color showed the group of neonatal metabolite profile, which is preceded by metabolite profile subgroups. Abbreviations: SDS, standard deviation scores; BMI, body mass index; Lyso.PC.e, alkyl-lysophosphatidylcholines; Lyso.PC.e_S, saturated Lyso.PC.e; and Lyso.PC.e_MUS, mono-unsaturated Lyso.PC.e.

We observed the statistical interaction of offspring sex with overall and saturated Lyso.PC.e (FDR *p*-values < 0.05). Among girls only, maternal continued smoking during pregnancy was associated with lower neonatal overall, mono-unsaturated and saturated Lyso.PC.e concentrations (FDR *p*-values < 0.05) (Figure 2A). Overall, these associations were not explained by birth outcomes (data not shown). Among boys only, maternal smoking during the first trimester only was associated with lower neonatal saturated Lyso.PC.e concentrations, but this association did not survive multiple testing correction (Figure 2B).

### 3.4. Sensitivity Analyses

First, we did not observe associations of exposure to paternal tobacco smoking or environmental smoking with neonatal metabolite profiles (data not shown). Second, the associations of maternal smoking during pregnancy with neonatal metabolite profiles were not explained by first trimester blood folate concentrations (data not shown).

## 4. Discussion

Fetal tobacco exposure is associated with alterations in the neonatal phospholipid profile. Neonates exposed to tobacco in the first trimester only had lower neonatal mono-unsaturated PC.ae and Lyso.PC.e.18:0 concentrations, whilst neonates exposed to tobacco throughout pregnancy had lower neonatal mono-unsaturated Lyso.PC.a, Lyso.PC.e.16:0, and Lyso.PC.e.18:1 concentrations. This study reveals differences in the metabolic signatures of neonates exposed to tobacco during pregnancy, both in the first trimester only and continued, as compared to those not exposed using advanced metabolomics analysis.

Fetal tobacco exposure leads to early developmental adaptations, such as changes in the physiology, metabolism, and structure of various organ systems [51]. Maternal smoking during pregnancy is a major risk factor for preterm birth and low birth weight and seems to be associated with long-term cardiovascular, respiratory and metabolic health outcomes. The metabolite profile shows the activity and status of various physiological functions; for example, how cell metabolism generates energy and biosynthetic precursors for growth and development of the embryo and the fetus [52]. We hypothesized that tobacco exposure during pregnancy, both in the first trimester only and continued, leads to metabolic adaptations in the unborn child, which might predispose individuals to cardio-metabolic disease in later life. Insight into the metabolic developmental adaptations in response to fetal tobacco exposure may contribute to the understanding of pathways in early life that predispose to cardiovascular and metabolic disease in later life.

We observed that neonates exposed to tobacco in the first trimester only had lower mono-unsaturated PC.ae and Lyso.PC.e.C18:0 concentrations, whilst neonates exposed to tobacco throughout pregnancy had lower mono-unsaturated Lyso.PC.a, Lyso.PC.e.C16:0, and Lyso.PC.e.C18:1 concentrations. Dose-response associations showed the strongest effect estimates in neonates whose mothers continued smoking ≥5 cigarettes. Thus, our study showed that fetal tobacco exposure, both in the first trimester only and continued, is associated with alterations in the neonatal phospholipid profile. Since phospholipids play a key role in embryonic and fetal development, a critical period in which tissues and organs develop, lower phospholipid concentrations may be the result of undesirable structural and functional changes in the metabolism of the unborn child (i.e., cardiovascular, respiratory and metabolic maladaptation) [51,53]. Consistent with our findings, animal studies in intrauterine growth-restricted (IUGR) rats showed that fetal nicotine exposure was associated with alterations in the fetal metabolome [54,55]. A previous study reported that IUGR rats exposed to nicotine during pregnancy had lower fetal unsaturated lipids, glucose, pyruvate, glycerol, and amino acids (as valine, lysine, leucine, glutamate, glutamine, and arginine) concentrations, but higher fetal lactate concentrations in blood samples by gestational day 20 [54]. However, our study did not detect changes in neonatal amino acid concentrations. This lack of association might be explained by the small number of children born with fetal growth restriction in our study population (0.7%). Additionally, a previous in vitro study suggested that part of the fetal amino acid deficit induced by maternal smoking may be compensated by inducing new amino acid transport systems because smoking induces the formation of new trophoblastic transporters for the uptake of alpha-aminobutyric acid [56]. Another study reported that rat fetuses exposed to high-doses of nicotine had reduced lipid concentrations (i.e., total cholesterol and triglycerides) in blood samples by gestational day 20 [55]. Smoking-induced metabolic alterations in the fetus may cause long-term structural and functional changes in metabolism. However, little is known about phospholipid metabolism in the human fetus.

A human prospective study in Germany among 40 mother-infant pairs showed that smoking during pregnancy alters both the maternal and neonatal metabolome [13]. Partly in line with our findings, mothers who smoked in pregnancy had lower maternal PC and SM concentrations at 34 weeks’ gestation in blood samples, whereas full-term neonates exposed to tobacco in pregnancy had lower neonatal Carn.a and higher PC and SM concentrations at birth in cord blood samples [13]. These differences across that study and our study may be explained by differences between gestational ages and clinical and sociodemographic characteristics. Previous studies in adults reported sex-specific differences between smokers and nonsmokers in adult metabolome profiles [11]. A growing body of literature suggested sex-specific differences in fetal programming in response to environmental stress, which may result in sex-specific risk for later disease [5,6,7,8,9,10,14,15]. Prenatal tobacco exposure appears to alter the neonatal metabolome profile differently in girls and boys. Our findings suggest that girls continuously exposed to tobacco during pregnancy had lower neonatal metabolite Lyso.PC.e concentrations. These findings are in line with a human in vitro study in which hepatoblasts, derived from pluripotent stem cells, were exposed to tobacco smoke to model their influence on hepatocyte development [57]. Hepatocytes from females previously exposed to tobacco underwent apoptosis during the process of cell differentiation, whereas hepatocytes from males appeared to become necrotic when separated from the extracellular matrix [57]. As fetal liver receives 70% of its umbilical vein blood supply directly from the placenta and the maternal-fetal interface, the fetal liver may be more exposed to active smoking compounds such as nicotine, polycyclic aromatic hydrocarbons, and cadmium, protecting the fetus from harmful chemicals and controlling fetal growth [58]. Understanding how smoking early and during pregnancy influences fetal metabolism and development may help us unravel the long-term pathophysiology of tobacco-induced cardiovascular, endocrine and metabolic diseases.

We did not observe associations of exposure to paternal tobacco smoking or environmental smoking with neonatal metabolite profiles. These findings suggest that the associations of fetal exposure to tobacco smoke with neonatal metabolite profiles are based on direct maternal smoking only. However, future studies with larger numbers of subjects may enable the identification of potential smaller effect estimates related to the associations of passive maternal smoking on neonatal metabolite profiles.

**Figure 3:**
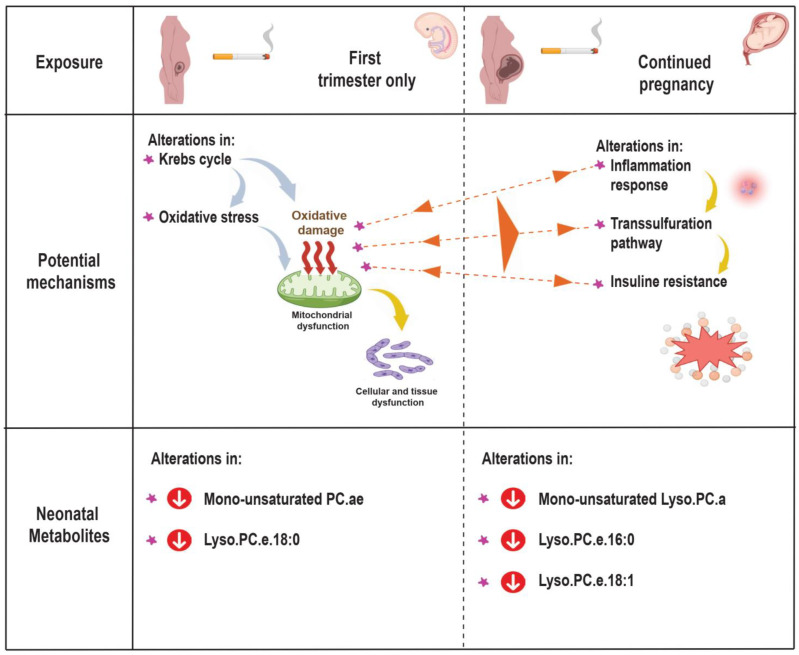
Potential mechanisms: Abbreviations: PC.ae, acyl-alkyl-phosphatidylcholines; Lyso.PC.a, acyl-lysophosphatidylcholines; and Lyso.PC.e, alkyl-lysophosphatidylcholines.

Several molecular and biological processes may represent the underlying mechanism between prenatal tobacco exposure and altered neonatal metabolome profiles. We observed that neonates exposed to tobacco in the first trimester only showed alterations in the metabolite ratios marking process of the Krebs cycle and oxidative stress, whereas those neonates exposed to tobacco throughout pregnancy showed alterations in the inflammatory response, transsulfuration and insulin resistance markers (Figure 3). In line with our study, previous studies focused on the maternal metabolome profile and metabolic pathways, and tried to explain the relationship between maternal smoking in pregnancy and adverse birth outcomes [59,60]. An untargeted metabolomics study in the United States (US) among 105 African American pregnant women suggested that both maternal cotinine concentrations and gestational age at birth were associated with altered maternal metabolic pathways in early and late pregnancy, such as oxidative stress, inflammatory response, insulin action and lipid metabolism [60]. Another untargeted metabolomics study in the US among 65 pregnant women showed that low-level maternal exposure to nicotine was associated with altered maternal metabolic pathways, such as aspartate and asparagine metabolism, arginine and proline metabolism, and methionine metabolism in second-trimester amniotic fluid samples [59]. In addition to this, animal studies showed that nicotine-induced IUGR in rats was associated with impaired glucocorticoid homeostasis in both mothers and fetuses [55]. Prenatal nicotine exposure in rats causes fetuses to be overexposed to maternal glucocorticoids and fetal hypothalamic-pituitary-adrenal axis development was inhibited [55]. Higher maternal glucocorticoid concentrations may promote alteration of the lipid and glucose metabolism and oxidative stress [55,61]. In the same way, another study showed that fetal nicotine exposure was associated with reduced postnatal mitochondrial structure and function, which may lead to impaired beta cell function, and subsequent dysglycemia in adult offspring [62]. Exposure to tobacco during critical periods of cell proliferation and differentiation can affect the developing fetus, and cause structural and functional alterations in cells, tissues, and organ systems. These alterations translate into an altered neonatal metabolic phenotype that may have lifelong consequences on body composition, cardiovascular, endocrine and metabolic health. Thus, prenatal tobacco exposure may stimulate and disrupt the fetal cardiovascular, endocrine and metabolic development, leading to an increased risk of obesity, hypertension, type 2 diabetes, and metabolic syndrome.

Increased inflammatory response, oxidative stress, and lipid metabolism remains poorly understood in the placenta and fetus despite its association with various pathological conditions, such as IUGR, cardiovascular, endocrine and metabolic disorders [52]. Our study suggests that fetal tobacco exposure is associated with metabolic changes in the neonate. Moreover, smoking during pregnancy may induce an adaptive or permanent response optimizing the growth and development of the key body organs to the detriment of other organs or systems [52]. Therefore, to prevent smoking in reproductive-aged women may constitute an important measure in public health.

### Strengths and Limitations

The strengths of our study are the prospective data collection from early pregnancy onwards, allowing the collection of information on tobacco per trimesters, as well as extensive information on potential confounders. Some limitations need to be discussed. First, the metabolomics data was only available in a subgroup of our multi-ethnic cohort, which consisted of Dutch participants only and relatively high educated and healthy participants as compared to the full cohort [63]. Ethnicity may influence the metabolome via both (epi) genetic and environmental factors [64]. By performing our study within an ethnic homogenous population, we reduced the risk of potential residual confounding or effect modification by ethnicity. However, our selected study population may affect the generalizability of our sample to the full cohort and the general population. Second, we adopted a targeted metabolomics approach, allowing us to optimize the quantification of metabolites of interest. However, relevant biological pathways might be missed. Third, the self-reported assessment of tobacco is a valid method, but misclassification may be possible. Participants may have underreported their tobacco use, which could potentially have led to an underestimation of the observed associations. Nonetheless, previous studies reported a high correlation between cotinine concentrations and reported smoking habits [60]. Finally, although we have adjusted for several sociodemographic and lifestyle factors, residual confounding might still be possible.

## 5. Conclusions

Our findings suggest that fetal tobacco exposure is associated with alterations in the neonatal phospholipid profile, mainly PC.ae, Lyso.PC.a, and Lyso.PC.e, and alterations in the metabolite ratios marking processes of Krebs cycle, oxidative stress, inflammatory response, lipid metabolism, and insulin resistance. Clearly, understanding the neonatal metabolome profile may yield new targets to predict, prevent and treat cardiovascular, endocrine and metabolic disease in later life.

## Figures and Tables

**Table 1:** Descriptive statistics of the study population.

		Maternal Smoking During		
	Total *N* = 828	Non-Smoking *n* = 631	First Trimester Only *n* = 77	Continued during Pregnancy *n* = 120
**Maternal characteristic**				
Age, years, mean (±SD)	31.4 (4.1)	31.7 (3.9)	30.9 (4.1)	30.2 (5.2) **
Educational level				
None/Primary (%)	2.3	1.3	5.2 *	5.8 **
Secondary (%)	35.4	31.5	33.8	56.7
Higher (%)	62.3	67.2	61.0	37.5
Pre-pregnancy body mass index, kg/m^2^, median (95% range)	22.5 (18.4–33.7)	22.6 (18.5–33.4)	22.1 (18.4–30.3)	22.3 (18.4–34.8)
Folate concentrations, nmol/L, median (95% range)	19.5 (6.1–39.6)	20.7 (6.3–40.5)	18.4 (7.3–35.0)	12.6 (5.7–33.3) **
Psychopathology score, median (95% range)	0.12 (0.00–0.79)	0.10 (0–0.63)	0.14 (0–0.71)	0.19 (0–1.69) **
Alcohol use				
Never drank in pregnancy (%)	32.2	34.1	15.6 **	33.3
First trimester only (%)	15.7	14.1	29.9	15.0
Continued drinking (%)	52.1	51.8	54.5	51.7
Paternal tobacco use, yes (%)	41.6	32.3	58.4 **	75.8 **
Environmental smoking				
No (%)	66.5	70.0	62.3	38.3 **
Occasionally (%)	21.3	19.8	28.6	20.8
Daily (%)	12.2	8.9	9.1	29.2
**Birth characteristics**				
Gestational age, weeks, median (95% range)	40.3 (36.7–42.3)	40.3 (37.0–42.3)	40.3 (36.7–42.3)	40.3 (36.6–42.4)
Premature birth (%)	2.9	2.5	3.9	4.2
Birth weight, grams, mean (±SD)	3555.0 (506.8)	3569.7 (488.8)	3601.8 (573.4)	3402.3 (532.8) **
Small for gestational age (%)	10.0	8.1	7.8	21.7 **
Low birth weight (%)	2.2	1.7	2.6	4.2 *
Female sex, yes (%)	46.1	46.8	51.9	39.2

Values are presented as means (±SD), medians (95% range), or percentages. Abbreviations: SD: standard deviation. * *p*-value < 0.05, ** *p*-value < 0.01. Premature birth, birth at gestational age <37 weeks. Small for gestational age at birth, gestational age-adjusted birth weight under 10th percentile. Low birth weight, birth weight less than 2500 g.

## Data Availability

The data that support the finding of this study are available from the corresponding author upon reasonable request. 3 Data available on request due to restrictions eg privacy or ethical. The data presented in this study are available on request from the corresponding author. The data are not publicly available due to consent restrictions.

## Supplementary Materials

The following supporting information can be downloaded at: https://www.mdpi.com/article/10.3390/metabo12111101/s1, Figure S1. flow chart of the study population; Text S1. metabolite measurements [65,66]; Table S1. metabolite ratios; Figure S2. directed acyclic graphic of the study; Table S2. cord blood metabolite concentrations; Table S3. non-response analysis; Figure S3. correlation between selected neonatal metabolite ratios; Figure S4. associations of maternal smoking during pregnancy with cord blood metabolite profile and ratios adjusted for birth weight; Figure S5. associations of maternal smoking during pregnancy with cord blood metabolite profile and ratios adjusted for gestational age; and Figure S6. associations of maternal smoking during pregnancy with cord blood metabolite profile and ratios adjusted for gestational age adjusted birth weight.

## Abbreviations

SGA	small size for gestational age
PC.ae	acyl-alkyl-phosphatidylcholines
PC.aa	diacyl-phosphatidylcholines
Carn.a	acyl-carnitines
PC	phosphatidylcholines
SM	sphingomyelins
LC/MS	liquid chromatography/mass spectrometry
NEFA	non-esterified fatty acids
Lyso.PC.a	acyl-lysophosphatidylcholines
Lyso.PC.e	alkyl-lysophosphatidylcholines
Carn	carnitines
JCBN	Joint Commission on Biochemical Nomenclature
QC	quality control
CV	coefficient of variation
SD	standard deviation
BCAA	branched-chain amino acids
AAA	aromatic amino acids
SDS	standard deviations scores
BMI	body mass index
BSI	Brief Symptom Inventory
FDR	false discovery rate
IUGR	intrauterine growth restriction
