# Supplementary material for: Fetal Exposure to Maternal Smoking and Neonatal Metabolite Profiles
Source: Metabolites. 2022 Nov 11;12(11):1101. doi: 10.3390/metabo12111101 (PMC9692997; doi:10.3390/metabo12111101)
Supplement: Supplementary file 1 [file metabolites-12-01101-s001.zip › metabolites-1979112-Supplementary.pdf]

**Supplementary material**

# Fetal exposure to maternal smoking and neonatal metabolite profiles

Kim N. Cajachagua-Torres MD[1,2], Sophia M. Blaauwendraad MD[1,2], Hanan El Marroun PhD[3,4], Hans Demmelmair[5], Berthold Koletzko[5], Romy Gaillard MD, PhD[1,2], Vincent W.V. Jaddoe MD PhD[1,2]

[1] The Generation R Study Group, Erasmus MC, University Medical Center Rotterdam, 3000 CA Rotterdam, The Netherlands

[2] The Department of Pediatrics, Erasmus MC, University Medical Center Rotterdam, 3000 CA Rotterdam, The Netherlands

[3] The Department of Child and Adolescent Psychiatry, Erasmus MC, Sophia Children's Hospital, 3000 CB Rotterdam, The Netherlands

[4] The Department of Psychology, Education and Child Studies, Erasmus School of Social and Behavioural Sciences, 3062 PA Rotterdam, The Netherlands

[5] Department of Pediatrics, Dr. von Huaner Children's Hospital, LMU University Hospitals, LMU—Ludwig Maximilians Universität Munich, 80539 Munich, Germany

**Correspondence to:** Vincent W.V Jaddoe, The Generation R Study Group (Na 29-08), Erasmus MC, University Medical Center Rotterdam, P.O. Box 2040, 3000 CA Rotterdam, the Netherlands; phone: +31 (0)10 704 3405; e-mail: v.jaddoe@erasmusmc.nl

**CONTENTS**


| | |
|---|---|
| **Figure S1.** | Flow chart of the study population |
| **Text S1.** | Metabolite measurements |
| **Table S1.** | Metabolite ratios |
| **Figure S2.** | Directed acyclic graphic of the study |
| **Table S2.** | Cord blood metabolite concentrations |
| **Table S3.** | Non-response analysis |
| **Figure S3.** | Correlation between selected neonatal metabolite ratios |
| **Figure S4.** | Associations of maternal smoking during pregnancy with cord blood metabolite profile and ratios adjusted for birth weight |
| **Figure S5.** | Associations of maternal smoking during pregnancy with cord blood metabolite profile and ratios adjusted for gestational age |
| **Figure S6.** | Associations of maternal smoking during pregnancy with cord blood metabolite profile and ratios adjusted for gestational age adjusted birth weight |

**Figure S1.** Flow chart of the study population.

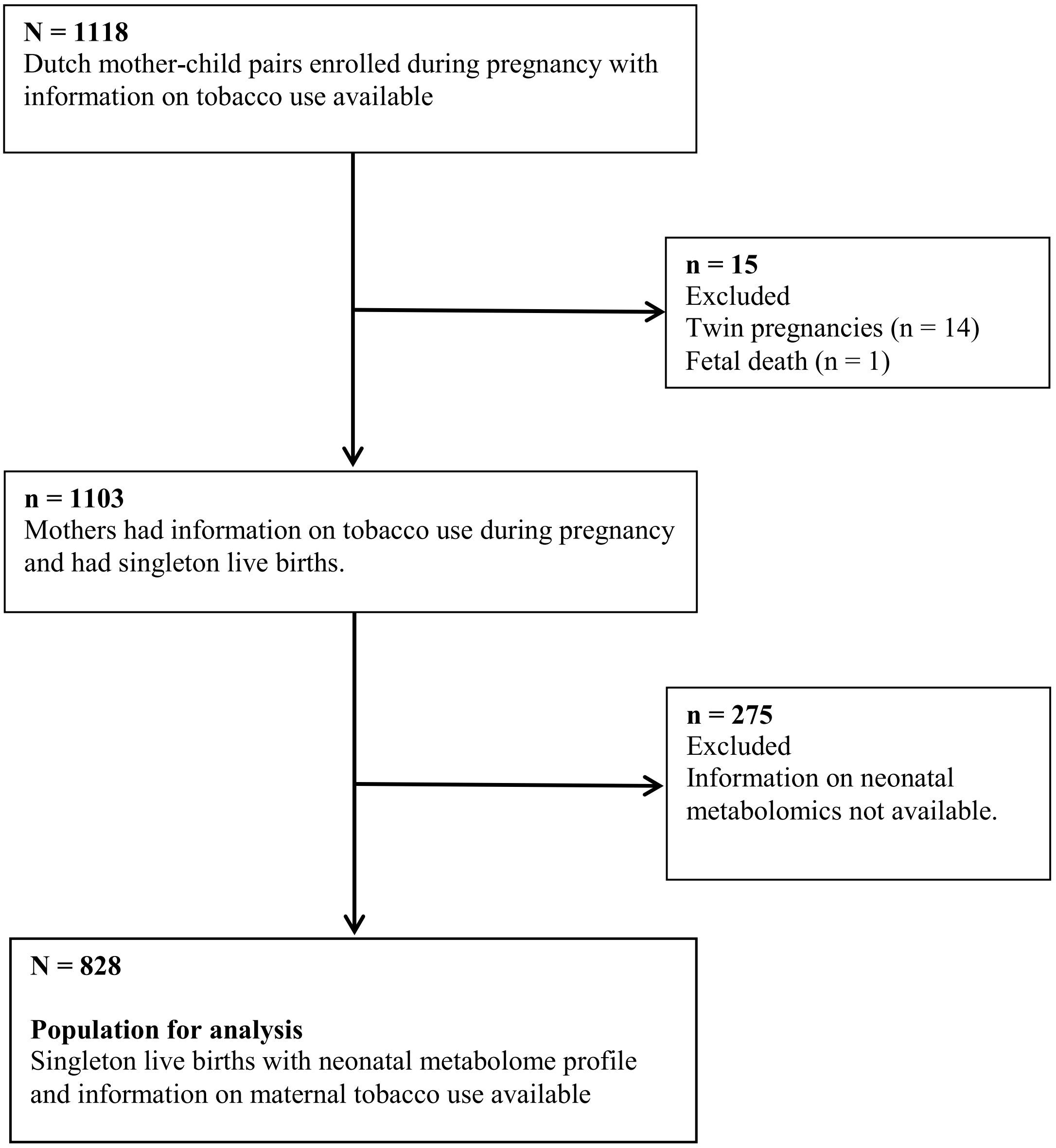

**Text S1.** Metabolite measurements.

A targeted metabolomics approach was performed at LMU Munich to determinate serum concentrations (µmol/L) of amino acids, non-esterified acids (NEFA), phospholipids and Carn, as described previously [1-5]. Proteins of 50 µL serum were precipitated by adding 450 µL methanol following internal standards: labeled amino acid standards set A (NSK-A-1, Cambridge Isotope Laboratories (CIL), USA), 15N2-L-asparagine (NLM-3286-0.25, CIL, USA), indole-D5-L-tryptophan (DLM-1092-0.5, CIL, USA), U-13C16-palmitic acid (CLM-409-MPTPK, CIL, USA), D3-acetyl-carnitine (DLM-754-PK, CIL, USA), D3-octanoyl-carnitine (DLM-755-0.01, CIL, USA) and D3-palmitoyl-carnitine (DLM-1263-0.01, CIL, USA), tridecanoyl-2-hydroxy-sn-glycero-3-phosphocholine (855476, Avanti Polar Lipids, USA) and 1,2-dimyristoyl-sn-glycero-3-phosphocholine (850345, Avanti Polar Lipids, USA) [6]. If sample volume was less than optimal, the concentrations were corrected by the respective factor. Sample volumes less than 25 µL were not used and considered missing. After centrifugation, the supernatant was split into aliquots. Amino acids were analyzed by liquid chromatography tandem mass spectrometry (LC-MS/MS) as described previously [3]. An aliquot of the supernatant was used for the derivatization to amino acids butylester with hydrochloric acid in 1-butanol. After evaporation, the residues were dissolved in water/methanol (80:20; (v/v)) with 0.1% formic acid. The samples were analyzed with 1100 high-performance liquid chromatography (HPLC) system (Agilent, Waldbronn, Germany) equipped with 150 x 2.1 mm, 3.5 µm particle size C18 HPLC column (X-Bridge, Waters, Milford, USA) and 0.1% heptafluorobutyric acid as and ion pair reagent in the mobile phases A and B (A: water, B: methanol). Mass spectrometric (MS) detection was performed with an API2000 tandem mass spectrometer (AB Sciex, Darmstadt, Germany) equipped with an atmospheric pressure chemical ionization (APCI) source operating in positive ion ionization mode. IUPAC-IUB Nomenclature was used for notation of the amino acids (1984).

NEFA, phospholipids and Carn were measured with a 1200 SL HPLC system (Agilent, Waldbronn, Germany) coupled to a 4000QTRAP tandem mass spectrometer from AB Sciex (Darmstadt, Germany) [4,7]. NEFA were analyzed by injection of the supernatant to a LC-MS/MS operating in negative electrospray ionization (ESI) mode where they were separated by gradient elution on a 100 x 3.0 mm, 1.9 µm particle size Purusuit UPS Diphenyl column from Varian (Darmstadt, Germany) using 5 mM ammonium acetate in water as mobile phase A and acetonitrile/ isopropanol (80:20; (v/v)) as mobile phase B. NEFA species were quantified using GLC-85 reference standard mixture (Nu-Chek Prep, USA). phospholipids were analyzed by flow-injection analysis (FIA) with LC-MS/MS coupled with ESI [8]. The system was run in positive ionization mode with 5% water in isopropanol as mobile phase A and 5% water in methanol as mobile phase B. The analysis was performed for diacyl-phosphatidylcholines (PC.aa), acyl-alkyl-phosphatidylcholines (PC.ae), acyl- lysophosphatidylcholines (Lyso.PC.a), alkyl-lysophosphatidylcholines (Lyso.PC.e) and sphingomyelines (SM)). Carn (Free carnitine (Free Carn) and acyl-carnitines (Carn.a)) were analyzed by flow-injection analysis of the supernatant into a LC-MS/MS system using an isocratic elution with 76% isopropanol, 19% methanol and 5% water. The mass spectrometer was equipped with electrospray ionization and operated in positive ionization mode. phospholipid and acyl-Carn were quantified using aliquots of a commercial available lyophilized control plasma (ClinChek®, Recipe, Germany), where the concentrations have been determined by AbsoluteIDQ p150 Kit from Biocrates®, a previous published LC-MS/MS method [9] and by in-house quantification with various standards. The analytical process was controlled and post-processed by Analyst 1.6.1. and R Software [6]. The analytical technique used is capable of determining the total number of total bonds, but not the position of the double bonds and the distribution of the carbon atoms between fatty acid side chains. We used the following notation for NEFA, phospholipids and Carn.a: X:Y, where X denotes the length of the carbon chain, and Y the number of double bonds. The ‘a’ denotes an acyl chain bound to the backbone via an ester bond (‘acyl-’) and the ‘e’ represents an ether bond (‘alkyl-’).”

**References**


1. Hellmuth, C.; Lindsay, K.L.; Uhl, O.; Buss, C.; Wadhwa, P.D.; Koletzko, B.; Entringer, S. Association of maternal prepregnancy BMI with metabolomic profile across gestation. *International Journal of Obesity* **2017**, *41*, 159-169, doi:10.1038/ijo.2016.153.
2. Jaddoe, V.W.V.; van Duijn, C.M.; van der Heijden, A.J.; Mackenbach, J.P.; Moll, H.A.; Steegers, E.A.P.; Tiemeier, H.; Uitterlinden, A.G.; Verhulst, F.C.; Hofman, A. The Generation R Study: design and cohort update until the age of 4 years. *Eur J Epidemiol* **2008**, *23*, 801, doi:10.1007/s10654-008-9309-4.
3. Harder, U.; Koletzko, B.; Peissner, W. Quantification of 22 plasma amino acids combining derivatization and ion-pair LC–MS/MS. *Journal of Chromatography B* **2011**, *879*, 495-504, doi:10.1016/j.jchromb.2011.01.010.
4. Hellmuth, C.; Weber, M.; Koletzko, B.; Peissner, W. Nonesterified Fatty Acid Determination for Functional Lipidomics: Comprehensive Ultrahigh Performance Liquid Chromatography–Tandem Mass Spectrometry Quantitation, Qualification, and Parameter Prediction. *Analytical Chemistry* **2012**, *84*, 1483-1490, doi:10.1021/ac202602u.
5. Blaauwendraad, S.M.; Voerman, E.; Trasande, L.; Kannan, K.; Santos, S.; Ruijter, G.J.G.; Sol, C.M.; Marchioro, L.; Shokry, E.; Koletzko, B.; et al. Associations of maternal bisphenol urine concentrations during pregnancy with neonatal metabolomic profiles. *Metabolomics* **2021**, *17*, 84, doi:10.1007/s11306-021-01836-w.
6. Hellmuth, C.; Uhl, O.; Standl, M.; Demmelmair, H.; Heinrich, J.; Koletzko, B.; Thiering, E. Cord Blood Metabolome Is Highly Associated with Birth Weight, but Less Predictive for Later Weight Development. *Obesity Facts* **2017**, *10*, 85-100, doi:10.1159/000453001.
7. Uhl, O.; Fleddermann, M.; Hellmuth, C.; Demmelmair, H.; Koletzko, B. Phospholipid Species in Newborn and 4 Month Old Infants after Consumption of Different Formulas or Breast Milk. *PloS one* **2016**, *11*, e0162040-e0162040, doi:10.1371/journal.pone.0162040.
8. Rauschert, S.; Uhl, O.; Koletzko, B.; Kirchberg, F.; Mori, T.A.; Huang, R.-C.; Beilin, L.J.; Hellmuth, C.; Oddy, W.H. Lipidomics Reveals Associations of Phospholipids With Obesity and Insulin Resistance in Young Adults. *The Journal of Clinical Endocrinology & Metabolism* **2016**, *101*, 871-879, doi:10.1210/jc.2015-3525.
9. Uhl, O.; Glaser, C.; Demmelmair, H.; Koletzko, B. Reversed phase LC/MS/MS method for targeted quantification of glycerophospholipid molecular species in plasma. *Journal of Chromatography B* **2011**, *879*, 3556-3564, doi:10.1016/j.jchromb.2011.09.043.

**Table S1.** Metabolite ratios.

| | | |
|---|---|---|
| a) | ∑PC.aa/∑PC.ae | reflecting oxidative stress |
| b) | PC.aa.C42:5/PC.ae.C36:0 | reflecting oxidative stress |
| c) | Asn/Asp<br>Gln/Glu | as indicators for anaplerosis or replenishing of Krebs cycle metabolites |
| d) | Pro/Glu | as marker of proline metabolism |
| e) | ∑Lyso.PC.a/∑PC.aa | as a lipid biomarker of inflammation |
| f) | (LysoPC.a.C18:1 + Lyso.PC.a.C18:2)/∑PC.aa | as an anti-inflammatory biomarker |
| g) | Carn.a.C2:0/Carn.a.C18:0 | as markers of fatty acid β-oxidation |
| h) | Met/Cys | as a marker of transsulfuration pathway |
| i) | Val/PC.ae.C32:2 | as a marker of insulin resistance |

**Figure S2.** Directed acyclic graphic of the study.

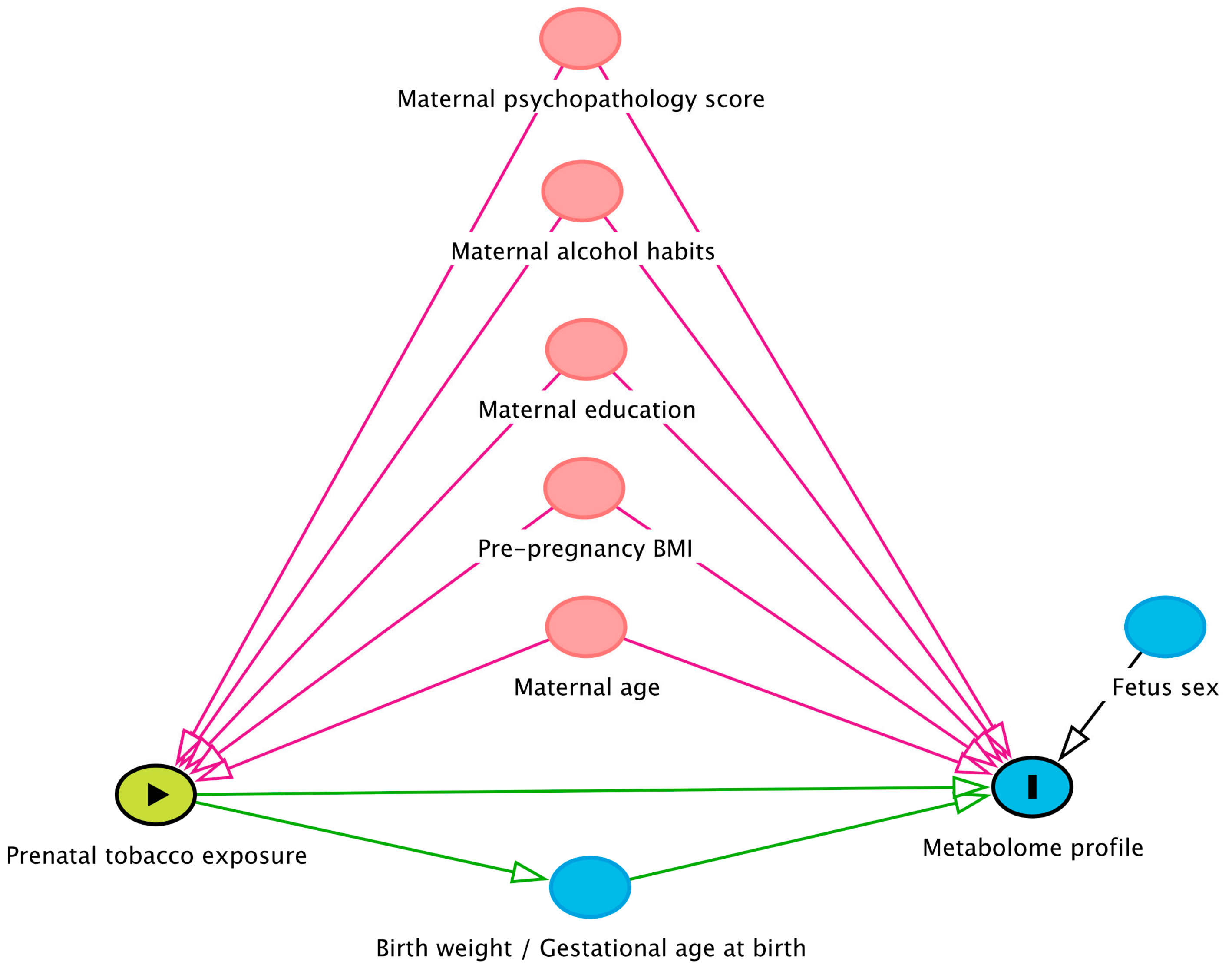

**Table S2.** Cord blood metabolite concentrations (N = 828).

| Neonatal metabolite profile | µmol/L, median (95%) |
|---|---|
| **Amino acids (AA)** | 3863.99 (2516.72, 5342.86) |
| BCAA | 480.56 (311.22, 710.87) |
| AAA | 257.05 (162.25, 368.40) |
| Essential AA | 1469.32 (997.85, 2034.21) |
| Non-essential AA | 2403.63 (1512.78, 3407.27) |
| **Ala** | 587.15 (329.51, 1013.83) |
| **Arg** | 76.15 (30.78, 130.57) |
| **Asn** | 56.80 (33.80, 95.61) |
| **Asp** | 40.84 (21.69, 80.8) |
| **Cit** | 13.92 (8.01, 23.96) |
| **Gln** | 443.01 (233.6, 824.05) |
| **Glu** | 196.67 (98.51, 404.77) |
| **Gly** | 326.11 (211.13, 488.26) |
| **His** | 132.92 (78.32, 211.73) |
| **Ile** | 74.78 (44.29, 115.3) |
| **Leu** | 144.20 (88.77, 228.63) |
| **Lys** | 340.08 (217.09, 579.05) |
| **Met** | 33.02 (19.82, 52.87) |
| **Orn** | 125.00 (71.36, 208.07) |
| **Phe** | 105.76 (65.36, 157.88) |
| **Pro** | 193.5 (121.04, 342.79) |
| **Trp** | 74.99 (45.17, 124.43) |
| **Ser** | 160.46 (94.61, 298.67) |
| **Thr** | 270.32 (153.46, 454.51) |
| **Tyr** | 75.21 (46.14, 115.58) |
| **Val** | 260.25 (169.64, 384.01) |
| **Cys** | 16.81 (7.11, 40.76) |
| | |
| **Non-esterified fatty acids (NEFA)** | 189.19 (81.76, 403.67) |
| Saturated NEFA | 90.52 (36.89, 188.43) |
| Mono-unsaturated NEFA | 56.14 (20.93, 133.31) |
| Poly-unsaturated NEFA | 43.69 (19.73, 91.46) |
| **NEFA.14:0** | 7.08 (2.45, 17.14) |
| **NEFA.14:1** | 1.53 (0.36, 4.33) |
| **NEFA.15:0** | 1.04 (0.35, 2.54) |
| **NEFA.16:0** | 66.60 (27.10, 140.36) |
| **NEFA.16:1** | 9.85 (2.96, 28.7) |
| **NEFA.16:2** | 0.46 (0.18, 1.15) |
| **NEFA.17:0** | 1.09 (0.49, 2.27) |
| **NEFA.17:1** | 0.52 (0.14, 1.36) |
| **NEFA.17:2** | 0.03 (0.00, 0.09) |
| **NEFA.18:0** | 13.97 (3.61, 30.46) |
| **NEFA.18:1** | 42.24 (16.73, 97.61) |
| **NEFA.18:2** | 23.94 (9.24, 56.78) |
| **NEFA.18:3** | 2.06 (0.51, 5.88) |
| **NEFA.19:1** | 0.15 (0.06, 0.32) |
| **NEFA.20:1** | 0.42 (0.16, 0.83) |

| | |
|---|---|
| **NEFA.20:2** | 0.55 (0.24, 1.11) |
| **NEFA.20:3** | 1.76 (0.77, 3.85) |
| **NEFA.20:4** | 8.13 (3.55, 15.71) |
| **NEFA.20:5** | 0.28 (0.09, 0.78) |
| **NEFA.22:3** | 0.13 (0.07, 0.25) |
| **NEFA.22:4** | 0.62 (0.34, 1.19) |
| **NEFA.22:5** | 0.74 (0.35, 1.52) |
| **NEFA.22:6** | 4.15 (1.85, 8.67) |
| **NEFA.24:0** | 0.17 (0.05, 0.32) |
| **NEFA.24:1** | 0.17 (0.06, 0.32) |
| **NEFA.24:2** | 0.09 (0.05, 0.18) |
| **NEFA.24:4** | 0.12 (0.06, 0.21) |
| **NEFA.24:5** | 0.10 (0.05, 0.19) |
| **NEFA.26:0** | 0.14 (0.05, 0.32) |
| **NEFA.26:1** | 0.09 (0.05, 0.16) |
| **NEFA.26:2** | 0.06 (0.03, 0.10) |
| | |
| **Diacyl-phosphatidylcholines (PC.aa)** | 750.21 (462.21, 1258.16) |
| Saturated PC.aa | 18.05 (10.75, 32.94) |
| Mono-unsaturated PC.aa | 129.10 (74.39, 236.20) |
| Poly-unsaturated PC.aa | 605.59 (374.78, 1010.75) |
| **PC.aa.C30:0** | 2.80 (1.43, 5.50) |
| **PC.aa.C30:3** | 0.13 (0.06, 0.26) |
| **PC.aa.C32:0** | 11.58 (6.29, 21.85) |
| **PC.aa.C32:1** | 9.99 (4.81, 22.07) |
| **PC.aa.C32:2** | 0.69 (0.07, 1.90) |
| **PC.aa.C32:3** | 0.30 (0.10, 0.60) |
| **PC.aa.C34:1** | 98.44 (57.24, 178.82) |
| **PC.aa.C34:2** | 67.48 (38.77, 130.41) |
| **PC.aa.C34:3** | 2.52 (1.21, 5.28) |
| **PC.aa.C34:4** | 0.42 (0.19, 0.84) |
| **PC.aa.C34:5** | 0.05 (0.01, 0.11) |
| **PC.aa.C36:0** | 1.10 (0.44, 2.11) |
| **PC.aa.C36:1** | 19.61 (11.20, 35.30) |
| **PC.aa.C36:2** | 39.07 (22.35, 70.08) |
| **PC.aa.C36:3** | 66.29 (34.99, 124.57) |
| **PC.aa.C36:4** | 143.39 (79.60, 239.36) |
| **PC.aa.C36:5** | 4.59 (2.29, 10.27) |
| **PC.aa.C36:6** | 0.24 (0.09, 0.51) |
| **PC.aa.C38:0** | 1.47 (0.66, 3.03) |
| **PC.aa.C38:2** | 2.85 (1.04, 5.97) |
| **PC.aa.C38:3** | 42.67 (22.22, 78.41) |
| **PC.aa.C38:4** | 100.76 (59.51, 173.68) |
| **PC.aa.C38:5** | 22.15 (12.71, 38.86) |
| **PC.aa.C38:6** | 69.42 (36.62, 132.21) |
| **PC.aa.C40:0** | 0.51 (0.21, 1.05) |
| **PC.aa.C40:1** | 0.22 (0.05, 0.46) |
| **PC.aa.C40:2** | 0.14 (0.03, 0.37) |
| **PC.aa.C40:3** | 0.41 (0.14, 0.90) |

| | |
|---|---|
| **PC.aa.C40:4** | 2.90 (1.53, 5.60) |
| **PC.aa.C40:5** | 6.81 (3.42, 14.16) |
| **PC.aa.C40;6** | 30.18 (14.16, 58.59) |
| **PC.aa.C42:0** | 0.58 (0.26, 1.11) |
| **PC.aa.C42:5** | 0.35 (0.15, 0.67) |
| **PC.aa.C43:6** | 1.73 (0.86, 3.37) |
| **PC.aa.C44:12** | 0.30 (0.15, 0.58) |
| **Acyl-alkyl-phosphatidylcholines (PC.ae)** | 73.29 (47.28, 126.67) |
| Saturated PC.ae | 12.04 (7.22, 20.92) |
| Mono-unsaturated PC.ae | 8.54 (5.11, 15.97) |
| Poly-unsaturated PC.ae | 52.7 (32.93, 92.1) |
| **PC.ae.C30:0** | 0.23 (0.08, 0.47) |
| **PC.ae.C32:0** | 2.34 (1.30, 4.49) |
| **PC.ae.C32:1** | 2.07 (1.06, 4.26) |
| **PC.ae.C32:2** | 0.44 (0.20, 0.97) |
| **PC.ae.C34:0** | 0.80 (0.40, 1.74) |
| **PC.ae.C34:1** | 3.70 (2.03, 7.45) |
| **PC.ae.C34:2** | 2.42 (1.39, 4.80) |
| **PC.ae.C34:3** | 1.09 (0.54, 2.34) |
| **PC.ae.C34:4** | 0.07 (0.02, 0.18) |
| **PC.ae.C36:0** | 0.54 (0.27, 1.09) |
| **PC.ae.C36:1** | 1.75 (0.95, 3.43) |
| **PC.ae.C36:2** | 2.01 (1.14, 3.94) |
| **PC.ae.C36:3** | 1.90 (1.01, 3.69) |
| **PC.ae.C36:4** | 8.61 (5.10, 15.93) |
| **PC.ae.C36:5** | 6.41 (3.53, 12.60) |
| **PC.ae.C38:0** | 0.95 (0.44, 1.85) |
| **PC.ae.C38:2** | 0.49 (0.14, 1.15) |
| **PC.ae.C38:3** | 1.66 (0.84, 3.17) |
| **PC.ae.C38:4** | 7.23 (4.24, 12.42) |
| **PC.ae.C38:5** | 7.46 (4.41, 13.63) |
| **PC.ae.C38:6** | 3.31 (1.98, 6.23) |
| **PC.ae.C40:0** | 7.1 (3.76, 12.86) |
| **PC.ae.C40:1** | 0.69 (0.25, 1.39) |
| **PC.ae.C40:2** | 0.43 (0.05, 1.24) |
| **PC.ae.C40:3** | 0.47 (0.17, 1.06) |
| **PC.ae.C40:4** | 1.81 (0.96, 3.38) |
| **PC.ae.C40:5** | 1.53 (0.85, 2.89) |
| **PC.ae.C40:6** | 2.13 (1.16, 4.04) |
| **PC.ae.C42:1** | 0.28 (0.11, 0.55) |
| **PC.ae.C42:3** | 0.28 (0.08, 0.64) |
| **PC.ae.C42:4** | 0.53 (0.21, 1.10) |
| **PC.ae.C42:5** | 1.10 (0.48, 2.33) |
| **PC.ae.C42:6** | 0.90 (0.41, 1.80) |
| **Acyl-lysophosphatidylcholines (Lyso.PC.a)** | 144.26 (82.65, 226.65) |
| Saturated Lyso.PC.a | 92.59 (54.22, 150.47) |
| Mono-unsaturated Lyso.PC.a | 17.81 (9.13, 31.49) |

| | |
|---|---|
| Poly-unsaturated Lyso.PC.a | 32.38 (16.86, 56.46) |
| **Lyso.PC.a.C14:0** | 3.25 (1.57, 5.68) |
| **Lyso.PC.a.C16:0** | 73.43 (42.26, 119.54) |
| **Lyso.PC.a.C16:1** | 4.73 (2.25, 8.84) |
| **Lyso.PC.a.C18:0** | 15.32 (9.4, 25.71) |
| **Lyso.PC.a.C18:1** | 13.09 (6.78, 23.11) |
| **Lyso.PC.a.C18:2** | 12.00 (5.62, 22.32) |
| **Lyso.PC.a.C18:3** | 0.29 (0.08, 0.66) |
| **Lyso.PC.a.C20:3** | 3.83 (1.73, 7.38) |
| **Lyso.PC.a.C20:4** | 13.32 (6.69, 24.72) |
| **Lyso.PC.a.C20:5** | 0.27 (0.06, 0.65) |
| **Lyso.PC.a.C22:6** | 2.43 (1.09, 4.66) |
| | |
| **Alkyl-lysophosphatidylcholines (Lyso.PC.e)** | 1.65 (0.84, 3.00) |
| Saturated Lyso.PC.e | 1.38 (0.66, 2.61) |
| Mono-unsaturated Lyso.PC.e | 0.28 (0.12, 0.48) |
| **Lyso.PC.e.C16:0** | 0.55 (0.27, 1.02) |
| **Lyso.PC.e.C18:0** | 0.84 (0.31, 1.70) |
| **Lyso.PC.e.C18:1** | 0.28 (0.12, 0.48) |
| | |
| **Sphingomyelines (SM)** | 220.81 (134.43, 377.50) |
| Mono-unsaturated SM | 106.7 (66.78, 181.17) |
| Poly-unsaturated SM | 112.86 (65.02, 203.72) |
| **SM.a.C30:1** | 0.13 (0.04, 0.28) |
| **SM.a.C32:1** | 2.97 (1.62, 5.20) |
| **SM.a.C32:2** | 0.45 (0.22, 0.86) |
| **SM.a.C33:1** | 2.18 (1.18, 3.92) |
| **SM.a.C34:1** | 54.69 (33.74, 96.86) |
| **SM.a.C34:2** | 11.20 (6.19, 20.05) |
| **SM.a.C35:1** | 1.66 (0.92, 3.11) |
| **SM.a.C36:1** | 19.51 (11.01, 32.63) |
| **SM.a.C36:2** | 12.52 (6.70, 23.42) |
| **SM.a.C36:3** | 0.36 (0.14, 0.76) |
| **SM.a.C37:1** | 0.95 (0.45, 1.83) |
| **SM.a.C38:2** | 6.06 (2.88, 14.02) |
| **SM.a.C38:3** | 0.20 (0.07, 0.33) |
| **SM.a.C39:1** | 1.35 (0.61, 2.68) |
| **SM.a.C39:2** | 0.46 (0.18, 0.83) |
| **SM.a.C40:2** | 12.03 (5.33, 25.09) |
| **SM.a.C40:5** | 0.26 (0.13, 0.51) |
| **SM.a.C41:1** | 4.01 (2.10, 7.70) |
| **SM.a.C41:2** | 3.42 (1.57, 6.88) |
| **SM.a.C42:1** | 18.00 (10.58, 32.71) |
| **SM.a.C42:2** | 35.42 (19.33, 65.21) |
| **SM.a.C42:3** | 18.09 (8.71, 35.56) |
| **SM.a.C42:4** | 6.83 (3.66, 12.08) |
| **SM.a.C42;6** | 2.78 (1.44, 5.32) |
| **SM.a.C43:1** | 0.95 (0.49, 1.88) |
| **SM.a.C43:2** | 1.27 (0.65, 2.39) |

| | |
|---|---|
| **SM.a.C44:6** | 1.41 (0.60, 2.54) |
| **SM.e.C36:2** | 0.24 (0.11, 0.47) |
| **SM.e.C38:3** | 0.06 (0.02, 0.15) |
| **SM.e.C40:5** | 0.22 (0.09, 0.42) |
| **Free Carn** | 16.57 (9.89, 27.67) |
| **Acyl-carnitine (Carn.a)** | 5.86 (3.56, 10.12) |
| Short-chain Carn.a | 4.47 (2.45, 8.49) |
| Medium-chain Carn.a | 0.52 (0.29, 0.88) |
| Long-chain Carn.a | 0.86 (0.50, 1.34) |
| **Carn.a.C10:0** | 0.09 (0.04, 0.19) |
| **Carn.a.C10:1** | 0.08 (0.04, 0.15) |
| **Carn.a.C12:0** | 0.10 (0.05, 0.18) |
| **Carn.a.C14:1** | 0.05 (0.02, 0.12) |
| **Carn.a.C14:2** | 0.04 (0.01, 0.08) |
| **Carn.a.C15:0** | 0.04 (0.02, 0.07) |
| **Carn.a.C16:0** | 0.16 (0.09, 0.28) |
| **Carn.a.C16:0.Oxo** | 0.02 (0.01, 0.04) |
| **Carn.a.C16:1** | 0.11 (0.05, 0.20) |
| **Carn.a.C16:2** | 0.03 (0.02, 0.07) |
| **Carn.a.C18:0** | 0.09 (0.05, 0.15) |
| **Carn.a.C18:1** | 0.09 (0.05, 0.17) |
| **Carn.a.C18:2** | 0.07 (0.03, 0.12) |
| **Carn.a.C18:2.OH** | 0.02 (0.01, 0.04) |
| **Carn.a.C2:0** | 3.75 (1.93, 7.37) |
| **Carn.a.C20:0** | 0.03 (0.02, 0.05) |
| **Carn.a.C20:1** | 0.00 (0.00, 0.00) |
| **Carn.a.C20:3** | 0.06 (0.03, 0.10) |
| **Carn.a.C20:4** | 0.00 (0.00, 0.01) |
| **Carn.a.C3:0** | 0.31 (0.17, 0.61) |
| **Carn.a.C3:0.DC** | 0.11 (0.04, 0.32) |
| **Carn.a.C4:0** | 0.14 (0.08, 0.26) |
| **Carn.a.C5:0** | 0.14 (0.06, 0.32) |
| **Carn.a.C6:0** | 0.05 (0.02, 0.12) |
| **Carn.a.C6:0.OH** | 0.04 (0.02, 0.10) |
| **Carn.a.C8:0** | 0.06 (0.02, 0.13) |
| **Carn.a.C8:1** | 0.05 (0.02, 0.12) |
| **Carn.a.C9:0** | 0.02 (0.01, 0.04) |

Values are presented as medians (95% range) of neonatal metabolites concentration in cord blood (μmol/L)

**Table S3.** Non-response analysis.

| | **Participants with information on neonatal metabolomics n = 828** | **Participants without information on neonatal metabolomics n = 275** | **p-value*** |
|---|---|---|---|
| **Maternal characteristic** | | | |
| Age, years, mean (±SD) | 31.4 (4.1) | 31.5 (4.3) | 0.71 |
| Missing (%) | - | - | |
| Educational level | | | |
| None/Primary (%) | 2.3 | 2.2 | 0.98 |
| Secondary (%) | 35.3 | 35.3 | |
| Higher (%) | 62.1 | 60.4 | |
| Missing (%) | 0.4 | 2.2 | |
| Maternal alcohol use | | | |
| Never during pregnancy (%) | 31.3 | 31.3 | 0.99 |
| First trimester only (%) | 15.2 | 14.9 | |
| Continued pregnancy (%) | 51.1 | 49.8 | |
| Missing (%) | 2.4 | 4.0 | |
| Pre-pregnancy BMI, $kg/m^2$, median (95% range) | 22.5 (18.4 – 33.5) | 22.6 (18.5 – 37.8) | 0.46 |
| Missing (%) | 12.2 | 8.4 | |
| Psychopathology score, median (95% range) | 0.1 (0.0 – 0.7) | 0.1 (0.0 – 0.7) | 0.75 |
| Missing (%) | 6.5 | 6.2 | |
| **Birth characteristic** | | | |
| Female sex, yes (%) | 46.1 | 53.5 | 0.03 |
| Missing (%) | - | 0.4 | |
| Gestational age, weeks, median (95% range) | 40.3 (36.7 – 42.3) | 40.1 (32.8 – 42.4) | 0.08 |
| Birth weight, gram, mean (±SD) | 3549.2 (506.6) | 3407.7 (658.6) | <0.01 |
| Missing (%) | 0.1 | 0.4 | |

Values are means and standard deviation (±SD), medians (95% range), or percentages.
*Differences in subjects' characteristics between the groups were evaluated using T-test for the normally distributed continuous variables, Man Whitney-U test for the not-normally distributed continuous variables, and chi-square tests for proportions.
Abbreviations: SD: standard deviation

**Figure S3.** Correlations between selected neonatal metabolite ratios.

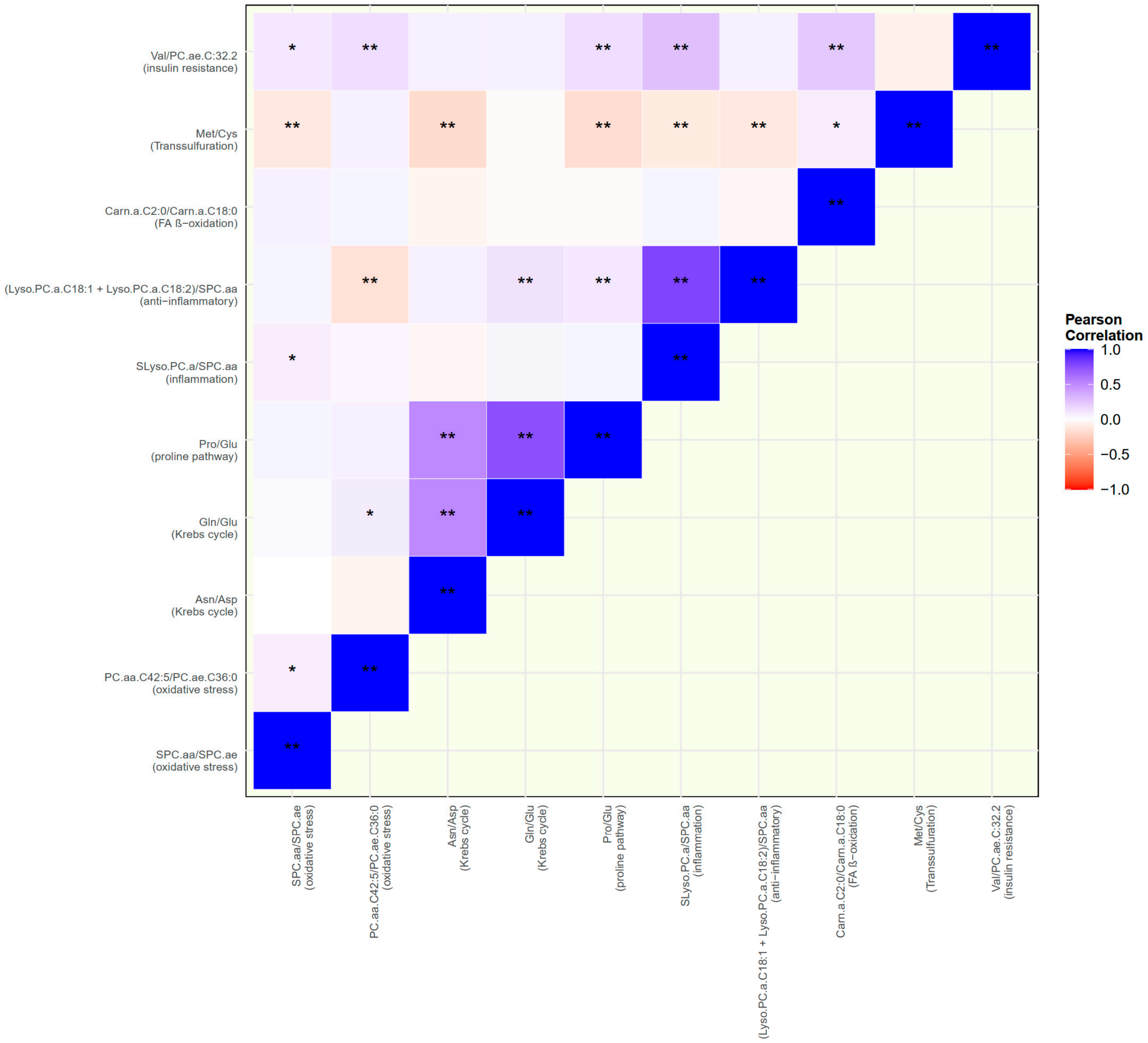


Values represent Pearson's correlation coefficient between selected neonatal metabolite ratios square root transformed. Abbreviations: PC.aa, diacyl-phosphatidylcholines; PC.ae, acyl-alkyl-phosphatidylcholines; Lyso.PC.a, acyl-lysophosphatidylcholines; Carn.a, acyl-carnitines; Asn/Asp, asparagine/aspartic acid; Gln/Glu, glutamine/glutamic acid; Pro/Glu, proline/glutamic acid; FA β-oxidation, Fatty acid β-oxidation; Met/Cys, methionine/cysteine; and Val, valine.
* p-value <0.05, ** p-value <0.01.

**Figure S4.** Associations of maternal smoking during pregnancy with cord blood metabolite profile and ratios adjusted for birth weight.

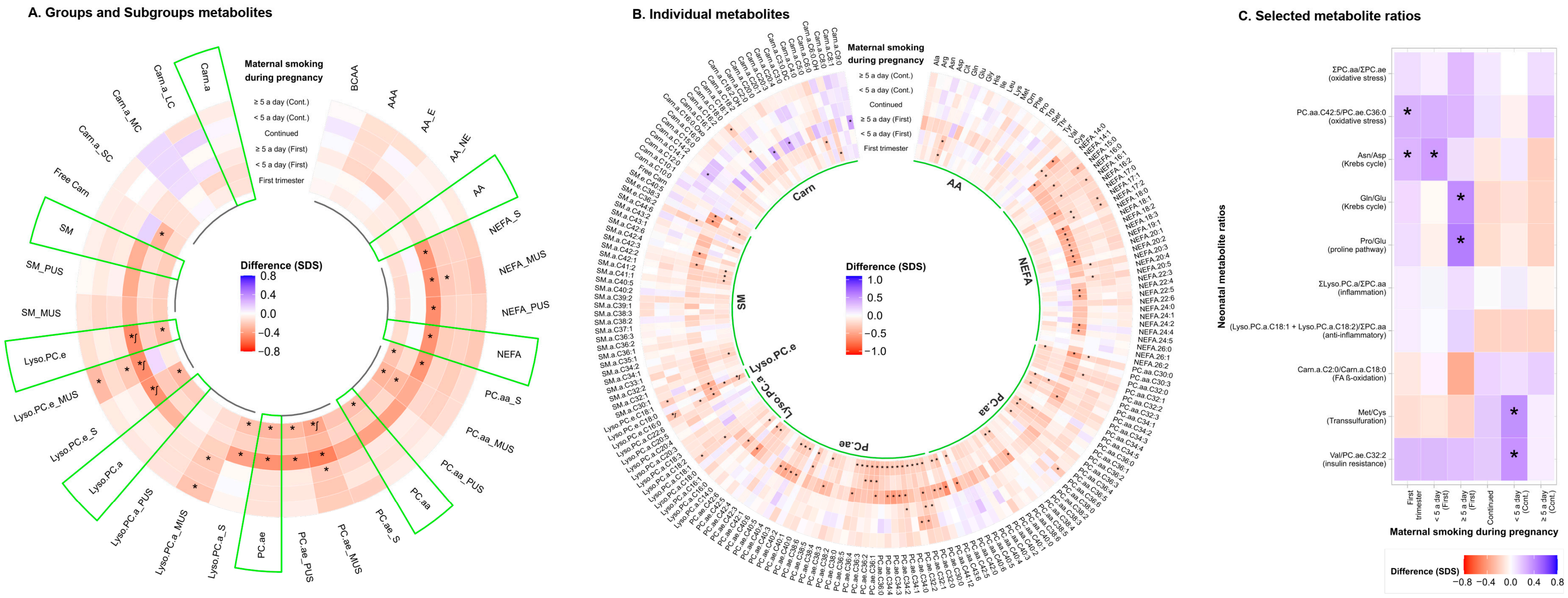


Values represent regression coefficients based on linear models that reflect the positive (blue) and negative (red) difference in neonatal cord blood metabolite concentrations and ratios in standard deviation scores in neonates whose mothers smoked any time of pregnancy compared to mothers who did not smoke. Models were adjusted for maternal age, educational level, alcohol consumption, pre-pregnancy BMI, psychopathology symptoms, fetal sex, and birth weight (birth model).

* p-value < 0.05.

ʃ Significant associations after FDR corrected p-value.

The sectors of green color showed group of neonatal metabolite profile, which is preceded by metabolite profile subgroups.

Abbreviations: SDS, standard deviation scores; BMI, body mass index; AA, amino acids; BCAA, branched-chain AA; AAA, aromatic AA; AA_E, essential AA; AA_NE, non-essential AA; NEFA, non-esterified fatty acids; NEFA_S, saturated NEFA; NEFA_MUS, mono-unsaturated NEFA; NEFA_PUS, poly-unsaturated NEFA; PC.aa, diacyl-phosphatidylcholines; PC.aa_S, saturated PC.aa; PC.aa_MUS, mono-unsaturated PC.aa; PC.aa_PUS, poly-unsaturated PC.aa; PC.ae, acyl-alkyl-phosphatidylcholines; PC.ae_S, saturated PC.ae; PC.ae_MUS, mono-unsaturated PC.ae; PC.ae_PUS, poly-unsaturated PC.ae; Lyso.PC.a, acyl-lysophosphatidylcholines; Lyso.PC.a_S, saturated Lyso.PC.a; Lyso.PC.a_MUS, mono-unsaturated Lyso.PC.a; Lyso.PC.a_PUS, poly-unsaturated Lyso.PC.a; Lyso.PC.e, alkyl-lysophosphatidylcholines; Lyso.PC.e_S, saturated Lyso.PC.e; Lyso.PC.a_MUS, mono-unsaturated Lyso.PC.e; SM, sphingomyelines; SM_MUS, mono-unsaturated SM; SM_PUS, poly-unsaturated SM; Free Carn, free carnitine; Carn.a, acyl-carnitines; Carn.a _SC, short-chain Carn.a; Carn.a _MC, medium-chain Carn.a; and Carn.a _LC, long-chain Carn.a; Asn/Asp, asparagine/aspartic acid; Gln/Glu, glutamine/glutamic acid; Pro/Glu, proline/glutamic acid; FA β-oxidation, Fatty acid β-oxidation; Met/Cys, methionine/cysteine; and Val, valine.

**Figure S5.** Associations of maternal smoking during pregnancy with cord blood metabolite profile and ratios adjusted for gestational age.

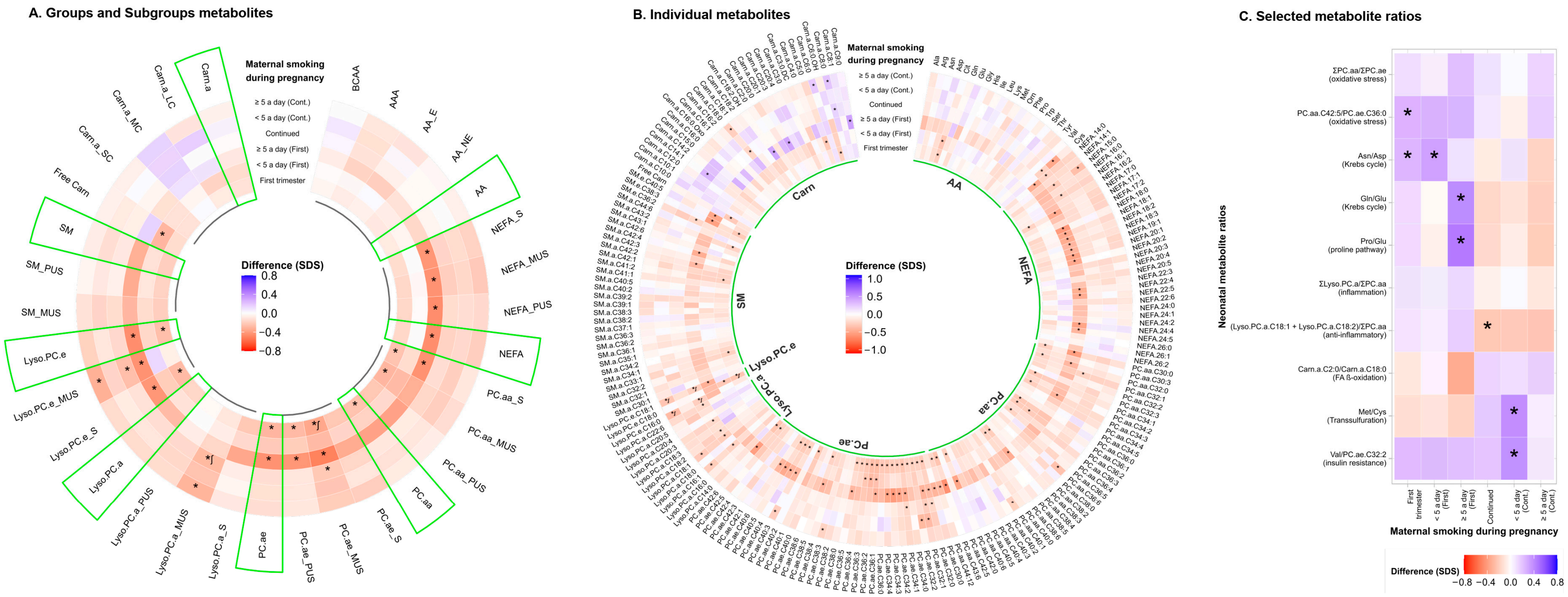


Values represent regression coefficients based on linear models that reflect the positive (blue) and negative (red) difference in neonatal cord blood metabolite concentrations and ratios in standard deviation scores in neonates whose mothers smoked any time of pregnancy compared to mothers who did not smoke. Models were adjusted for maternal age, educational level, alcohol consumption, pre-pregnancy BMI, psychopathology symptoms, fetal sex, and gestational age (birth model).

* p-value < 0.05.

ʃ Significant associations after FDR corrected p-value.

The sectors of green color showed group of neonatal metabolite profile, which is preceded by metabolite profile subgroups.

Abbreviations: SDS, standard deviation scores; BMI, body mass index; AA, amino acids; BCAA, branched-chain AA; AAA, aromatic AA; AA_E, essential AA; AA_NE, non-essential AA; NEFA, non-esterified fatty acids; NEFA_S, saturated NEFA; NEFA_MUS, mono-unsaturated NEFA; NEFA_PUS, poly-unsaturated NEFA; PC.aa, diacyl-phosphatidylcholines; PC.aa_S, saturated PC.aa; PC.aa_MUS, mono-unsaturated PC.aa; PC.aa_PUS, poly-unsaturated PC.aa; PC.ae, acyl-alkyl-phosphatidylcholines; PC.ae_S, saturated PC.ae; PC.ae_MUS, mono-unsaturated PC.ae; PC.ae_PUS, poly-unsaturated PC.ae; Lyso.PC.a, acyl-lysophosphatidylcholines; Lyso.PC.a_S, saturated Lyso.PC.a; Lyso.PC.a_MUS, mono-unsaturated Lyso.PC.a; Lyso.PC.a_PUS, poly-unsaturated Lyso.PC.a; Lyso.PC.e, alkyl-lysophosphatidylcholines; Lyso.PC.e_S, saturated Lyso.PC.e; Lyso.PC.a_MUS, mono-unsaturated Lyso.PC.e; SM, sphingomyelines; SM_MUS, mono-unsaturated SM; SM_PUS, poly-unsaturated SM; Free Carn, free carnitine; Carn.a, acyl-carnitines; Carn.a _SC, short-chain Carn.a; Carn.a _MC, medium-chain Carn.a; and Carn.a _LC, long-chain Carn.a; Asn/Asp, asparagine/aspartic acid; Gln/Glu, glutamine/glutamic acid; Pro/Glu, proline/glutamic acid; FA β-oxidation, Fatty acid β-oxidation; Met/Cys, methionine/cysteine; and Val, valine.

**Figure S6.** Associations of maternal smoking during pregnancy with cord blood metabolite profile and ratios adjusted for gestational age adjusted birth weight.

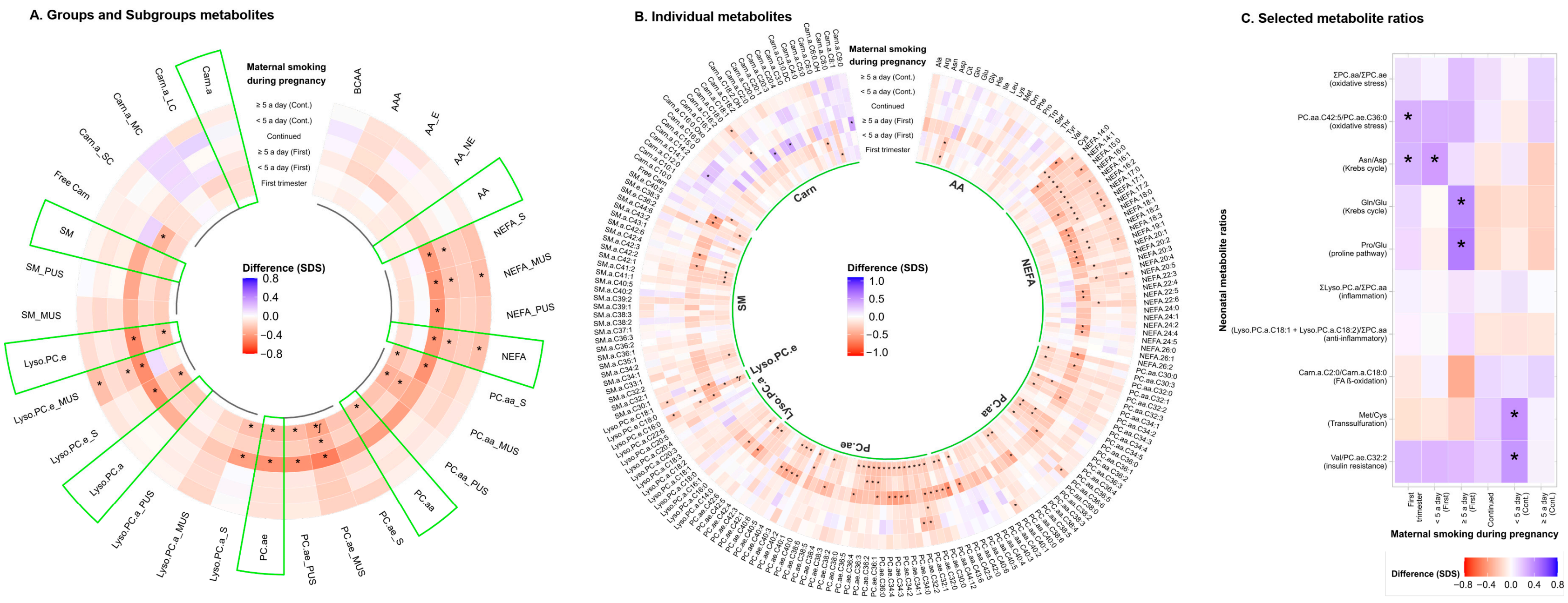


Values represent regression coefficients based on linear models that reflect the positive (blue) and negative (red) difference in neonatal cord blood metabolite concentrations and ratios in standard deviation scores in neonates whose mothers smoked any time of pregnancy compared to mothers who did not smoke. Models were adjusted for maternal age, educational level, alcohol consumption, pre-pregnancy BMI, psychopathology symptoms, fetal sex, and Niklasson standard (birth model).

* p-value < 0.05.

ʃ Significant associations after FDR corrected p-value.

The sectors of green color showed group of neonatal metabolite profile, which is preceded by metabolite profile subgroups.

Abbreviations: SDS, standard deviation scores; BMI, body mass index; AA, amino acids; BCAA, branched-chain AA; AAA, aromatic AA; AA_E, essential AA; AA_NE, non-essential AA; NEFA, non-esterified fatty acids; NEFA_S, saturated NEFA; NEFA_MUS, mono-unsaturated NEFA; NEFA_PUS, poly-unsaturated NEFA; PC.aa, diacyl-phosphatidylcholines; PC.aa_S, saturated PC.aa; PC.aa_MUS, mono-unsaturated PC.aa; PC.aa_PUS, poly-unsaturated PC.aa; PC.ae, acyl-alkyl-phosphatidylcholines; PC.ae_S, saturated PC.ae; PC.ae_MUS, mono-unsaturated PC.ae; PC.ae_PUS, poly-unsaturated PC.ae; Lyso.PC.a, acyl-lysophosphatidylcholines; Lyso.PC.a_S, saturated Lyso.PC.a; Lyso.PC.a_MUS, mono-unsaturated Lyso.PC.a; Lyso.PC.a_PUS, poly-unsaturated Lyso.PC.a; Lyso.PC.e, alkyl-lysophosphatidylcholines; Lyso.PC.e_S, saturated Lyso.PC.e; Lyso.PC.a_MUS, mono-unsaturated Lyso.PC.e; SM, sphingomyelines; SM_MUS, mono-unsaturated SM; SM_PUS, poly-unsaturated SM; Free Carn, free carnitine; Carn.a, acyl-carnitines; Carn.a _SC, short-chain Carn.a; Carn.a _MC, medium-chain Carn.a; and Carn.a _LC, long-chain Carn.a; Asn/Asp, asparagine/aspartic acid; Gln/Glu, glutamine/glutamic acid; Pro/Glu, proline/glutamic acid; FA β-oxidation, Fatty acid β-oxidation; Met/Cys, methionine/cysteine; and Val, valine.
